# Elucidating osseointegration in vivo in 3D printed scaffolds eliciting different foreign body responses

**DOI:** 10.1016/j.mtbio.2023.100771

**Published:** 2023-08-19

**Authors:** Dewei Qiu, Chuanliang Cao, Aruna Prasopthum, Zhenchang Sun, Shan Zhang, Hanwen Yang, Zhiyong Xu, Jun Tao, Fanrong Ai, Jing Yang

**Affiliations:** a Department of Orthopedic Surgery, The Second Affiliated Hospital of Nanchang University, Jiangxi, China; b School of Advanced Manufacturing, Nanchang University, Jiangxi, China; c Department of Oncology, The First Affiliated Hospital of Zhengzhou University, Henan, China; d School of Pharmacy, Walailak University, Thailand; e School of Pharmacy, University of Nottingham, UK; f Biodiscovery Institute, University of Nottingham, UK

**Keywords:** Biomaterials, 3D printing, Bone, Tissue engineering, Osseointegration, Foreign body response

## Abstract

Osseointegration between biomaterial and bone is critical for the clinical success of many orthopaedic and dental implants. However, the mechanisms of in vivo interfacial bonding formation and the role of immune cells in this process remain unclear. In this study, we investigated the bone-scaffold material interfaces in two different 3D printed porous scaffolds (polymer/hydroxyapatite and sintered hydroxyapatite) that elicited different levels of foreign body response (FBR). The polymer/hydroxyapatite composite scaffolds elicited more intensive FBR, which was evidenced by more FBR components, such as macrophages/foreign body giant cells and fibrous tissue, surrounding the material surface. Sintered hydroxyapatite scaffolds showed less intensive FBR compared to the composite scaffolds. The interfacial bonding appeared to form via new bone first forming within the pores of the scaffolds followed by growing towards strut surfaces. In contrast, it was previously thought that bone regeneration starts at biomaterial surfaces via osteogenic stem/progenitor cells first attaching to them. The material-bone interface of the less immunogenic hydroxyapatite scaffolds was heterogenous across all samples, evidenced by the coexistence of osseointegration and FBR components. The presence of FBR components appeared to inhibit osseointegration. Where FBR components were present there was no osseointegration. Our results offer new insight on the in vivo formation of bone-material interface, which highlights the importance of minimizing FBR to facilitate osseointegration for the development of better orthopaedic and dental biomaterials.

## Graphical abstract

**Figure:**
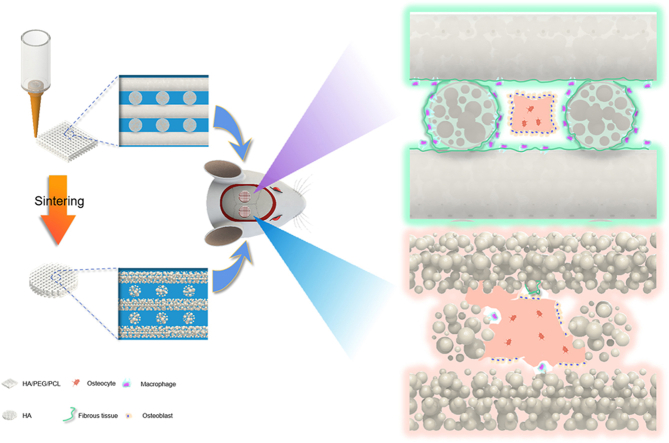

## 1 Introduction

The integration of biomaterials to host bone to form strong bonding is critical for the clinical success of many orthopaedic and dental implants. A strong bonding prevents excessive micromotion and loosening of the implanted biomaterial under physiological forces. This bonding has been named osseointegration and defined as the direct apposition of bone on implant surface without intervening connective/fibrous tissue [1,2]. Osseointegration was studied in the 1970s by Hench and co-workers who pioneered the development of bioglass which formed a bond with bone so strong that it could not be removed without breaking the bone [[3], [4], [5]]. A serial of studies was conducted to illustrate why bioglass formed such a strong bonding with bone. It became clear that the formation of a carbonated hydroxyapatite (CHA) layer on the surface of bioglass is part of the mechanism responsible for the strong bonding. The process of forming this CHA layer on bioglass is clear now [6]. In general, the dissolution of glass and precipitation of dissolved ions from the glass and its surrounding onto the glass surface is key in this process of forming a CHA layer. The formation of CHA also happens to various calcium phosphates [[7], [8], [9]].

However, the formation of a CHA layer alone is not sufficient to explain the strong bonding between materials and bone as it does not address the bonding between the CHA and bone. The osseointegration of the CHA layer to host bone was thought to involve protein adsorption, attachment of bone-forming stem/progenitor cells to the CHA layer, cell osteogenic differentiation and the excretion of bone extracellular matrix and its subsequent mineralization. Hench et al. hypothesized that since the CHA layer has calcium, silicon and phosphorus ion concentrations similar to that of normal bone in osteogenesis, osteoblasts recognize it as a surface on which to lay down collagen and mucopolysaccharides [3,10]. This hypothesized process has also been called “contact osteogenesis” [11]. This hypothesis is supported by in vitro studies where osteolineage cells were seeded directly on material surfaces [12,13]. However, in vivo evidence for this hypothesized process of osteogenic cells attaching to and subsequent new bone growing from biomaterial surfaces is sparse [6,11].

It is reasonable to assume that the first protein adsorption step will be similar in vivo given the immediate contact between implants and blood during surgery. However, the subsequent steps in which bone-forming stem/progenitor cells attach to biomaterials may not be the same in vivo compared to what has been observed in vitro. The main reason for this hypothesis is the inflammatory and wound healing responses associated with implanted biomaterials [14,15]. The surgical injury and biomaterial implantation induces an acute inflammation in which blood-borne immune cells are first responders and dominate the cell population at the site. This inflammatory response will evolve over time and eventually leads to foreign body response (FBR) [14]. The initial inflammation environment is also harsh (hypoxia, low pH, oxidative burst by granulocytes, high potassium and sodium concentrations [16]) for bone-forming stem/progenitor cells but not for some immune cells such as macrophages [17]. Therefore, the formation of osseointegration must be considered in the context of FBR. Although there have been studies in which transmission electron microscopy (TEM) has been employed as a main tool to characterize the ultrastructure of the material-bone interface where osseointegration is found [7,[18], [19], [20]], the overall status of the interface and the role of immune cells is still unclear. Our previous study showed new bone regeneration not from the biomaterial surfaces but away from biomaterial surfaces in the central region of the pores within 3D printed scaffolds [21]. Herein, we hypothesise that 1) the in vivo formation of biomaterial-bone bonding is via the growth of new bone towards biomaterial surfaces rather than growing from biomaterial surface; 2)The FBR has a local inhibitory effect on bone formation, and where direct bone apposition on biomaterial surface happens depends on the distribution of FBR components (macrophages, foreign body giant cells and fibrous tissue), hence a heterogenous bone-biomaterial interface is expected. We used a rat calvaria defect model and analyzed bone-biomaterial interfaces at different time points within 3D printed porous scaffolds of different materials. A suit of methods including histology, immunohistochemistry, electron microscopy was employed to elucidate the characteristics of the biomaterial-bone interfaces.

## 2 Materials and methods

### 2.1 Fabrication of scaffolds

Polycaprolactone (PCL, *M*_n_ = 80,000Da), poly(ethylene glycol) (PEG, *M*_n_ = 400Da) and hydroxyapatite microparticles were all purchased from Sigma-Aldrich. The PCL/PEG/HA (84 wt% HA) scaffolds was fabricated as previously described [21]. Briefly, PEG was dissolved in dichloromethane (DCM). HA microparticles were then added and mixed well. PCL was lastly added, and the mixture was stirred overnight. The mixture was loaded into a pneumatic extrusion 3D printer. At room temperature, a 260 μm diameter needle was used with extrusion pressures of 4–5.5 bar and printing speeds of 4 mm/s. All scaffolds were dried in room temperature air for 24 h to evaporate the DCM. The HA scaffolds were made by sintering the HA/PEG/PCL(84%HA) scaffolds in a furnace at 1400 °C for 3 h.

### 2.2 Scanning electron microscope (SEM) and X-ray diffraction (XRD)

SEM images were obtained using the Quanta200FEG scanning electron microscope (FEI,USA).The samples were coated with gold/palladium before imaging. For histological slices, the wax block slices were dewaxed with xylene, dried and coated with gold/palladium before imaging. XRD measurements of the scaffold powder were performed using XRD (D8 ADVANCE, Bruker, Germany).

### 2.3 Compression testing of scaffolds

Compression testing was performed with an electromechanical universal testing machine (CMT6104, MTS Systems, China) and a 5 kN load cell at a speed of 0.5 mm/min. Compressive modulus was calculated according to the slopes of compression stress-strain curves. The scaffolds used for compression testing were 10 mm × 10 mm × 10 mm (length × width × height).

### 2.4 In vitro testing

Cytotoxicity was evaluated using the indirect method. 1 g scaffold was added into 5 ml minimum essential medium (MEM, Biological Industries, Israel) without serum and incubated at 37 °C for 24 h. The scaffold-conditioned media was collected and stored at 4 °C. MC3T3-E1 (mouse osteoblastic cell line) were cultured in MEM supplemented with 10% fetal bovine serum (Biological Industries, Israel) and 1% penicillin-streptomycin (ThermoFisher Scientific, USA) in a humidified incubator at 37 °C and 5% CO_2._ 100 μl of MC3T3-E1 suspension were seeded in a 96-well plate at a density of 4 × 10^4^ cells ml^−1^ and cultured overnight. The adhered cells were then cultured with a mixed medium consisting of conditioned media and culture media (1:1) for 1,3, and 7 days. At different time points, 10 μl Cell Counting Kit-8 (CCK-8) reagent (Beyotime, Shanghai, China) was added to each well. After incubating for 2 h, the absorbance was measured by a microplate reader (Molecular Devices, USA) at 450 nm to assess cell viability.

The cytotoxicity of the scaffold was further verified by Dead/live cell staining. The calcein-AM/propidium iodide double stain kit (40747ES76, Yeasen Biotechnology, Shanghai, China) was used to assess cytotoxicity according to the manufacturer's instructions. 4 × 10^4^ MC3T3-E1 cells were seeded in a 24-well plate and cultured overnight. Scaffold conditioned media were added on day 1 and day 3 and cultured for 30 min before imaging for live and dead cells. Cells were imaged using a fluorescence microscope (TE2000, Nikon, Japan).

### 2.5 In vivo testing

All animal procedures are carried out in accordance with the guidelines for the care and use of experimental animals of Nanchang University and approved by the Animal Ethics Committee of Nanchang University. 11-week-old male Sprague Dawley rats (Changsha Tianqin Biotechnology Co., Ltd) were used to establish a calvarial defects of 5 mm each according to our previous method [21]. The HA/PEG/PCL scaffolds (5 mm diameter, 1 mm thick, 5 layers of struts) were soaked in ultra-pure water for 24 h and then dried. All scaffolds were sterilized with ethylene oxide before implantation. All animals were operated under sterile conditions. Before surgery, the rats were anesthetized by intraperitoneal injections of 10% chloral hydrate (0.3 ml per 100 g of body weight). Each rat was implanted with two different scaffolds (HA/PEG/PCL and HA), and the wound was closed with absorbable sutures. All the experimental rats were in good condition and no wound infection was found. At different times after implantation (1, 2, 4, 8 and 12 weeks), the rats were euthanized and the scaffolds with some surrounding tissues were retrieved for micro-CT, hematoxylin&eosin (H&E) staining, Masson's trichrome, Immunohistochemistry, Picrosirius Red staining.

### 2.6 Micro-CT analysis

The porosity of the scaffolds and bone volume were measured by using micro-CT. For implanted scaffolds, the retrieved specimens were fixed with 4% paraformaldehyde for 48 h, then scanned with custom-made Micro-CT (iSA μCT H010, Xidian University, China) with a tube voltage of 78 kV and a tube current of 100 μA.

### 2.7 Histological analysis

After CT scanning, the specimens were decalcified in 0.5 M EDTA decalcifying solution for 30 days with frequent changing of the solution every 2–3 days, and then cut into 5 μm-thick sections. The specimens were embedded in paraffin blocks, and transversal slices with 5 μm thickness were cut from the middle of each defect using a microtome. The slices were then dewaxed followed by hydration in ethanol series and water before hematoxylin and eosin staining. Masson staining took the same dewaxing and hydration steps as HE staining, and then the slices were stained using a masson dye kit. Sirius red staining also took the same dewaxing and hydration steps, and then the slices were stained in a Sirius scarlet dye solution for 8 min. For immunohistochemistry, the sections were dewaxed in the same way as before. It was then incubated in 3% hydrogen peroxide solution, then incubated with 3% bovine serum albumin to block endogenous peroxidase and non-specific binding, and then incubated with anti-CD163, anti-iNOS, and anti-TGF-β1 antibodies. The slices were then washed and incubated in the second antibody tagged with horseradish peroxidase, and then stained in 3,3′-diaminobenzidine chromogenic solution to make CD163, iNOS or TGF-β1 appear brown. All reagents used for histology were from Wuhan Servicebio Technology, China. The positive regions of CD163, iNOS, TGF-β1 were quantitatively analyzed by ImageJ.

### 2.8 Statistical analysis

Statistical analysis was performed using GraphPad Prism 8, and the data were expressed as the mean ± SD. Statistical differences were analyzed with Student's t-test (for only two groups) or one-way analysis of variance (ANOVA) and Tukey's post hoc multiple comparison test.

## 3 Results

**Fig. 1:**
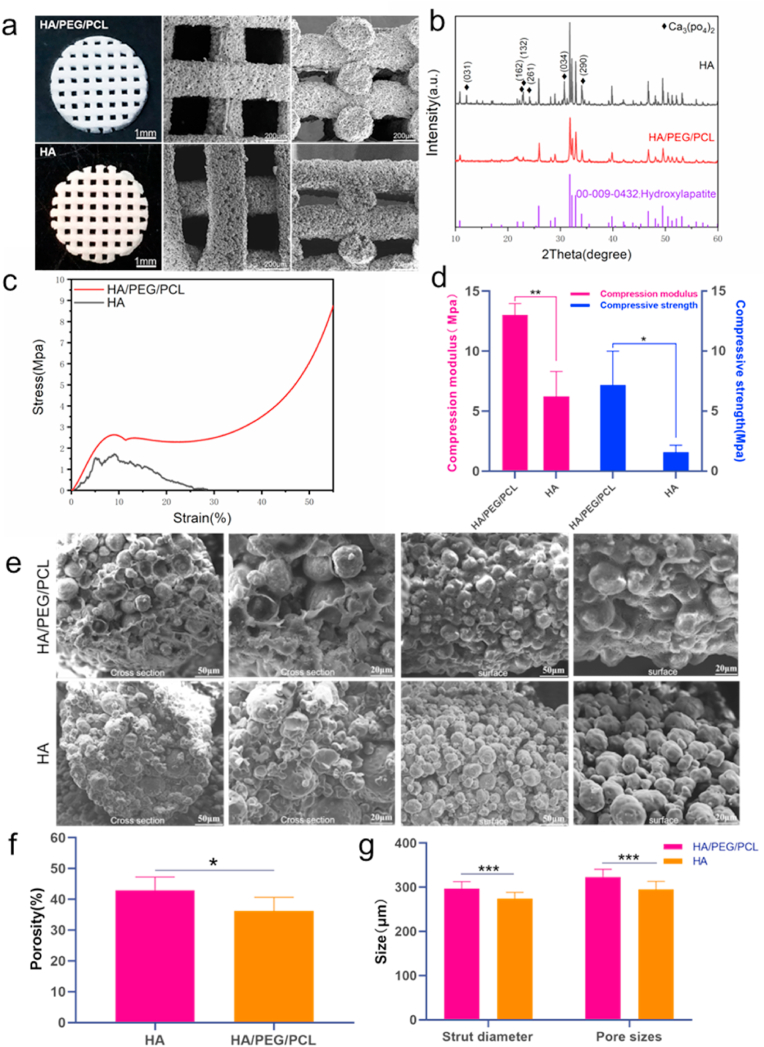
a) optical and SEM images of the HA/PEG/PCL and HA scaffolds; b) XRD graphs of the scaffold materials. Diamond shaped black dots represent peaks associated with Ca_3_PO4_2_; c) representative stress-strain curves of the two types of scaffolds; d) compressive modulus and strength of the scaffolds (n = 3). e) SEM images of the cross sections and strut surfaces of scaffolds. The HA/PEG/PCL scaffolds were immersed in water for 24 h to remove PEG before SEM. f) Porosity of HA/PEG/PCL and HA scaffolds (n = 5). g) The strut diameter and pore sizes of HA/PEG/PCL and HA scaffolds (n = 15). Data are represented as mean value ± SD. *P < 0.05, **P < 0.01, ***P < 0.001.

Scaffolds with the pore size (X–Y plane) of approximately 300 μm were fabricated. This pore size was selected based on previous studies which reported optimal pore sizes for bone regeneration [22]. HA scaffolds were made by sintering the HA/PEG/PCL scaffolds. The shape and dimensions were largely unchanged after sintering likely due to the high HA content in the scaffolds (Fig. 1a). The XRD graph showed the presence of tricalcium phosphate after sintering (Fig. 1b), which is a consequence of decomposition of HA after sintering at high temperature [23]. The particulate morphology of the HA particles was clearer for the sintered scaffolds which had the polymers being removed. HA/PEG/PCL scaffold surface showed relatively less gaps between the particles whilst sintered HA scaffold surface appeared relatively more porous. This was expected as PCL and PEG were removed during sintering. This increase of void within struts was also reflected in the increased matrix formation within the internal of struts after implantation. The porosity of HA scaffolds was statistically higher than that of the HA/PEG/PCL scaffolds (43% vs 36%). The sintered scaffold showed a slightly smaller strut diameter and pore size. The sintered scaffolds were much weaker compared to their composite counterparts according to the compression testing (Fig. 1c and d). However, as the implantation site (rat calvarial defects) was not load-bearing, most sintered HA scaffolds were able to maintain their shape integrity after the 12-week implantation.

**Fig. 2:**
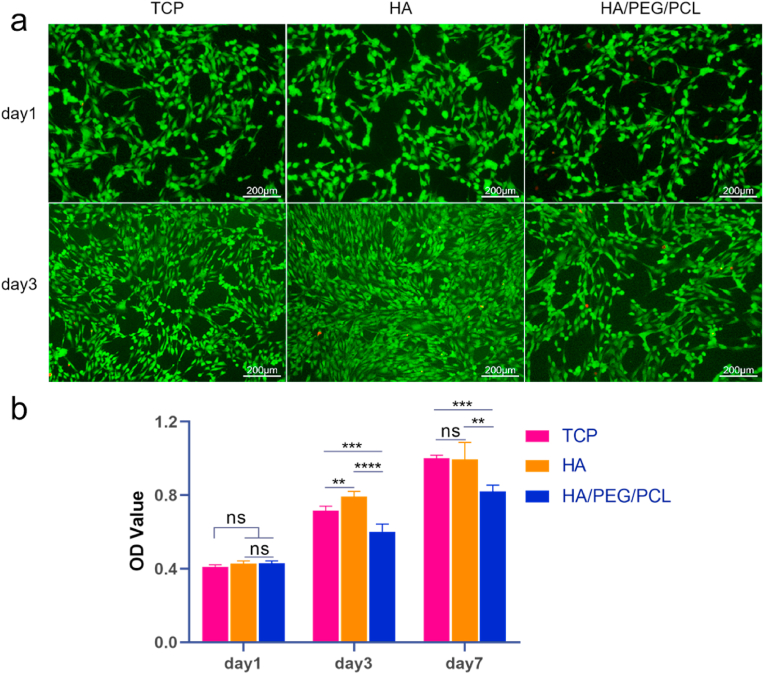
Cytotoxicity of the two different scaffolds. a) fluorescent images of MC3T3-E1 cells in different media (unconditioned and scaffold-conditioned) at day 1 and day 3 stained by Calcein-AM/PI (live/dead stating, green/red); b) the OD value (quantified by CCK-8 assay) of the three different cultures at different days. Data are represented as mean values ± SD (n = 5). *P < 0.05, **P < 0.01, ***P < 0.001,****P < 0.0001. TCP - tissue culture plastic.

To test cytotoxicity, MC3T3-E1 cells were cultured with the conditioned media of the two types of scaffolds. Live/dead staining showed minimum dead cells (stained red) at day 1 and day 3 (Fig. 2a). Cells proliferated in both cultures. However, the proliferation was lower at day 3 for the HA/PEG/PCL scaffold-conditioned media compared to that for sintered HA scaffolds (Fig. 2b). There was no further decrease of cell proliferation from day 3 to day 7. The leaching out of PEG in the composite scaffolds was possibly the cause of the lowered cell proliferation. The composite scaffolds were not incubated in water to remove PEG before being conditioned in media. Although PEG has been generally considered safe and widely used in pharmaceutical formulations and tissue engineering, the concentration and molecular weight of PEG are important factors for their negative effects on cells [24,25].

**Fig. 3:**
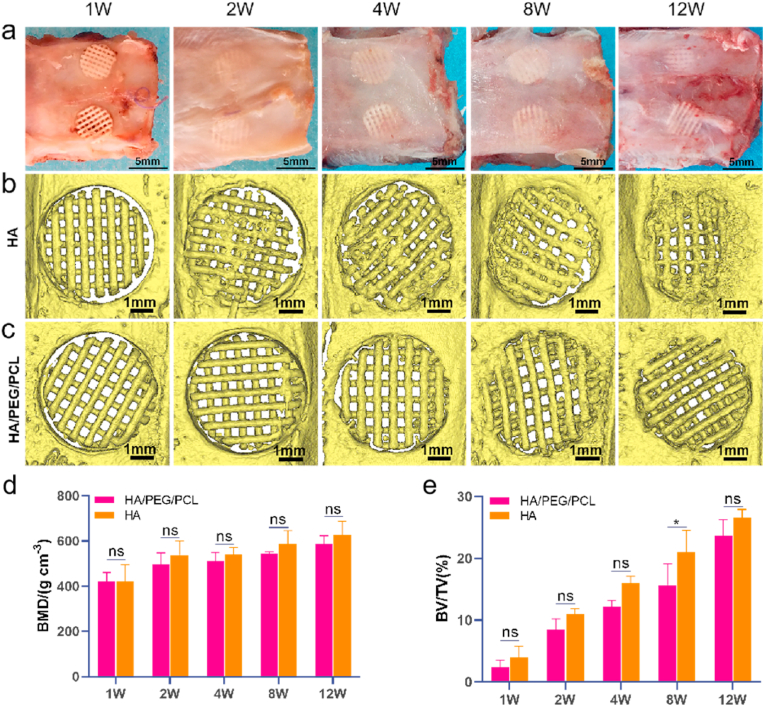
a) Gross view of the retrieved scaffolds at different times. b,c) MicroCT images of the implanted scaffolds at different times; d,e) Bone mineral density (BMD) and Bone volume/Total volume ratio (BV/TV). The volume of scaffolds was excluded for the quantification of BV. Data are represented as mean values ± SD (n = 3). *p < 0.05.

After studying cytotoxicity of these two different scaffolds in vitro, we then investigated bone regeneration and the interfaces between new bone and the scaffolds in vivo. Prior to implantation, the HA/PEG/PCL scaffolds were incubated in water for 24 h to remove PEG as our previous result showed that water incubation removed PEG without affecting scaffold mechanical properties [21]. The scaffolds were then air dried and sterilized by ethylene oxide before implantation. The gross views and microCT images of the retrieved scaffolds are shown in Fig. 3a&b. Both BMD and BV/TV increased over time for both types of scaffolds (Fig. 3d and e). There was no difference between the two types of scaffolds in bone mineral density. The BV/TV ratio was similar between the scaffold types except week 8. Scaffolds maintained their morphology over the 12-week implantation. No visible degradation was seen based on the μCT images, which was not surprising given the low degradation rates of both PCL and HA.

**Fig. 4:**
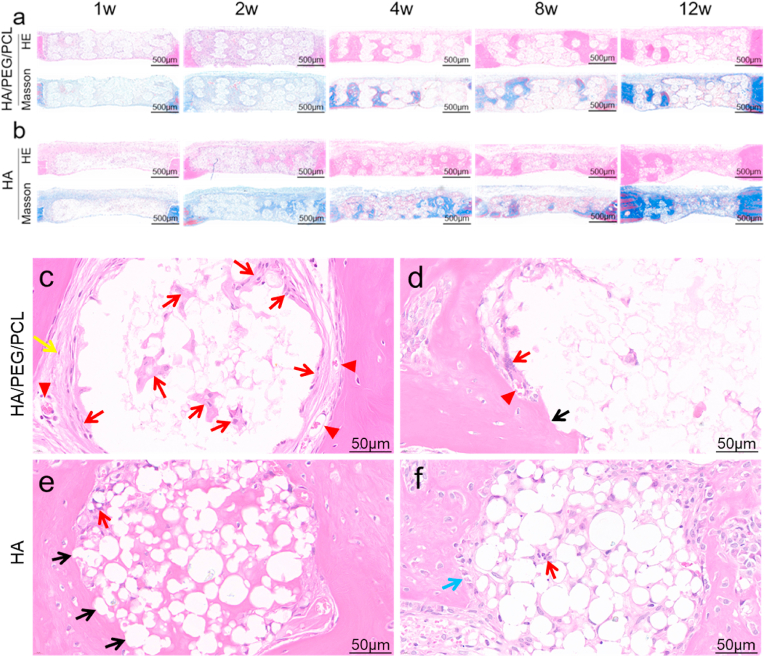
a,b) H&E and Masson staining of sagittal cross-sectional sections of scaffolds; c,d,e,f) high-magnification images of new bone–strut interfaces. Black arrow–osseointegration; red arrow–macrophages/FBGCs; yellow arrow–fibrous tissue; blue arrow–osteoblasts; red arrowhead–blood vessels.

Although the amount of bone regeneration within the two types of scaffolds was similar, histology revealed drastically different material-bone interfaces. The H&E and Masson staining images of the whole scaffolds are shown in Fig. 4a&b. Most strut surface of the HA/PEG/PCL scaffolds was covered by fibrous tissue which separated new bone from the biomaterial surfaces (Fig. 4c and supplementary figure 1). Osseointegration was only occasionally seen (Fig. 4d). Foreign body giant cells (FBGCs) were commonly found surrounding the struts (red arrows in Fig. 4c and d). Some FBGCs were visible within the strut, demonstrating the infiltration of macrophages into the inside of the struts. The high HA concentration caused these composite scaffolds to possess porous struts besides the macro-pores that were designed into the scaffolds during 3D printing. Some blood vessels were visible within the fibrous tissues that surround the struts.

In contrast, the sintered HA scaffolds showed much less fibrous tissue surrounding the scaffolds (Fig. 4e and f and supplementary figure 1). There was significantly more direct contact between bone and HA particles which was visible at the optical microscopic resolution level. FBGCs were also present on some areas of the HA strut surfaces as well as inside these struts (red arrows), but significantly less compared to the composite scaffolds (Supplementary figure 1). This observation demonstrates that the HA/PEG/PCL scaffold elicited a more intensive FBR compared to the HA scaffolds. The inside of the struts of the HA scaffolds also showed more matrix which was related to the removal of polymers and consequently more voids within the struts.

Histological images showed that bone regeneration started at locations away from material surface and subsequently grew towards them to form direct contacts. This is demonstrated by the relative locations of new bone and biomaterial. Eosin-stained new bone collagen (dense pink) appeared to surround the struts with or without osseointegration (Fig. 4c,d,e,f), which indicated the direction of bone growth towards biomaterial. In addition, where osseointegration appeared to about to form showed osteoblasts on new bone surface and osteoid between these cells and HA particles (blue arrow in Fig. 4d and supplementary figure 2), which also demonstrated the direction of new bone growth.

**Fig. 5:**
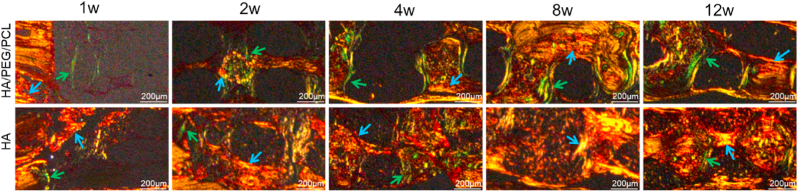
Picrosirius Red staining of collagen types within the two different scaffolds at different times. Collagen III and I appear green and orange, respectively. Green arrows - collagen type III; blue arrows - collagen type I.

To further demonstrate the difference in FBR surrounding the struts of the two types of scaffolds, picrosirius red staining which can differentiate type 1 and type III collagen under polarised light was used to characterize the types of collagens associated with the two scaffolding materials [26]. Previous studies have shown that during the process of fibrous capsule formation associated with implants, type III collagen was first secreted by fibroblasts then replaced by type I collagen [27]. Picrosirius red staining showed that there was more type III collagen present in the HA/PEG/PCL scaffold compared to the HA scaffolds (Fig. 5), which correlated with H&E staining results that showed more intensive FBR associated with the composite scaffolds.

**Fig. 6:**
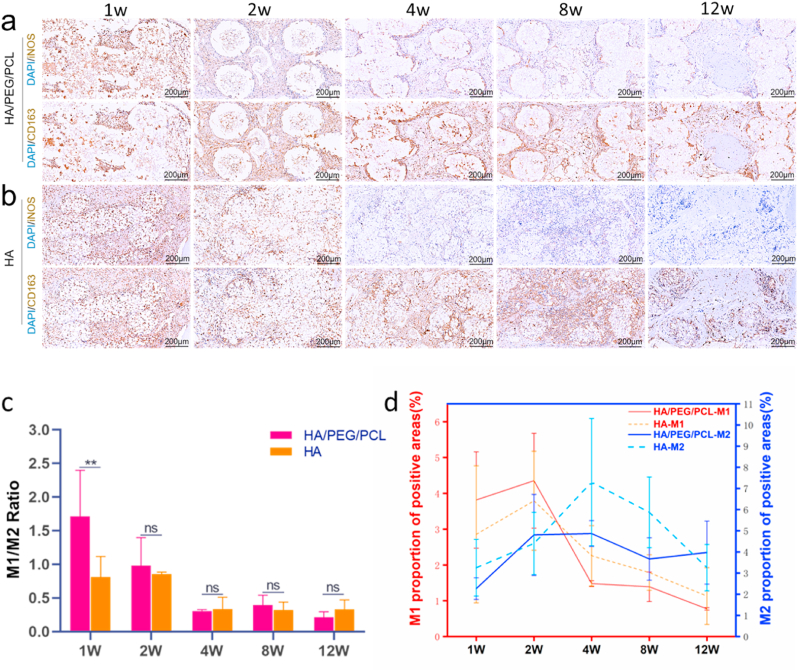
a, b) Immunohistochemistry images of M1 and M2 macrophages (brown colour); c) M1/M2 ratio over time; d) M1 and M2 level over time. M1 and M2 were quantified by positive area of M1(iNOS) and M2(CD163) markers. M1 and M2 markers were stained (brown colour) on adjacent microtome sections (5 μm apart). Data are represented as mean values ± SD (n = 3). **P < 0.01

As macrophages are perhaps the most important type of cells in FBR [28], these cells with different activation status were stained (iNOS–pro-inflammatory M1, CD163–anti-inflammatory M2) on microtome-sectioned slices. Although it is recognized that a spectrum of macrophage activation status exists rather than two opposing polarization status [29], the M1 versus M2 model is still helpful in characterizing the FBR in our study. Representative immunohistochemistry images of the two markers are shown in Fig. 6a&b. The M1/M2 ratio was initially (week 1) much higher for the HA/PEG/PCL scaffolds (Fig. 6c). This observation correlated to the findings in Fig. 4, Fig. 5 where more FBR was found for the composite scaffolds. There was little difference in M1/M2 ratio between the two types of scaffolds at later time points. The M1/M2 ration for both scaffolds decreased from week 1 to week 4 after which a stable ratio was established. Quantification of M1 and M2, respectively, showed that M1 level was higher at the first two weeks followed by a decrease to week 12 (Fig. 6d). M2 level seemed to peak around week 4 followed by a reduction for the HA scaffolds. The M2 level appeared to be more persistent for the composite scaffolds after 2 weeks.

These data suggested that the early pro-inflammatory condition played an important role in the evolution of FBR. It is well established nowadays that early inflammation is essential for removal of debris and dead cells, for promoting angiogenesis, and for recruiting cells to the fracture site [30]. However, unproportionally strong early (<72 h) pro-inflammatory reaction has been linked to decreased osteogenic differentiation (although correlation to fibrosis was not investigated) [31]. As both scaffolds showed similar reduction of inflammation (decreasing M1/M2 ratio), the increased level of fibrous tissue associated with the HA/PEG/PCL scaffolds was more likely due to the stronger early pro-inflammatory environment rather than more prolonged inflammation. Chronic activation of M2 can also lead to elevated fibrosis [32]. However, the level of M2 between the two types of scaffolds was not statistically different at various time points despite a seemingly more persistent of M2 for the composite scaffolds (supplementary figure 3).

**Fig. 7:**
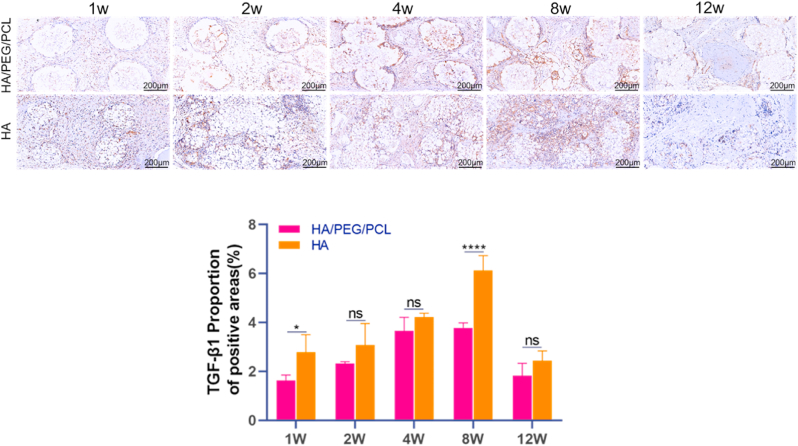
Representative immunohistochemistry images and quantification of TGF-β1 within the two different scaffolds over time. TGF-β1 appears brown in the images. Data are represented as mean ± SD (n = 3). *p < 0.05, ****P < 0.0001.

As TGF-β1 is secreted by M2 macrophages and FBGCs and it is a potent factor that promotes the differentiation of fibroblasts to myofibroblasts and fibrosis [33,34], the expression of this growth factor over time was quantified based on immunohistochemical staining. The amount of TGF-β1 increased till week 8 followed by a significant decrease till week 12 (Fig. 7). Although both scaffolds secreted similar level of TGF-β1 (except more for the HA scaffolds at week1 and 8), the level of fibrosis was much less for the HA scaffolds compared to the composite scaffolds. Interestingly, the BV/TV ratio was also higher for the HA scaffolds at week 8. Besides its role in promoting fibrosis, TGF-β1 also promotes the proliferation, chemotaxis, early differentiation of osteoprogenitor [35]. Our data suggested that the effector function of TGF-β1 in the HA scaffolds was likely more for promoting bone formation than for fibrosis.

**Fig. 8:**
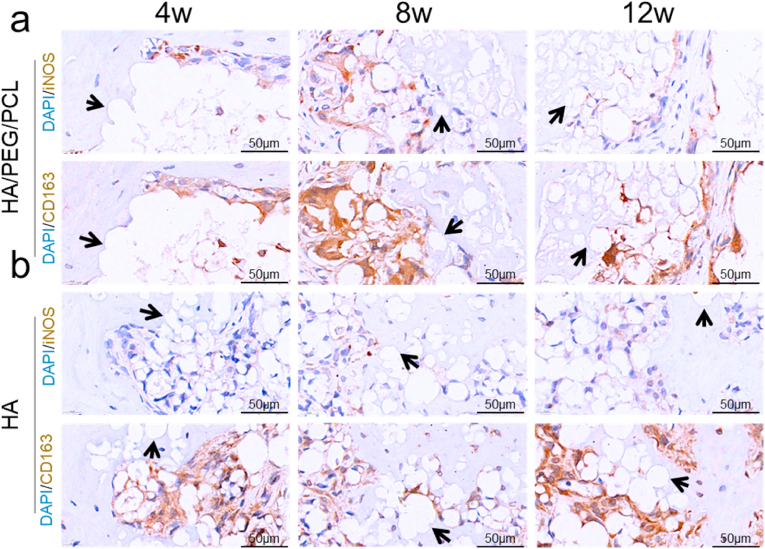
Immunohistochemistry of macrophages within the scaffolds. iNOS and CD163 were selected as the M1 and M2 marker, respectively. Cell nuclei were stained by DAPI(blue). iNOS and CD163 appear brown in the images. The iNOS and CD163 were stained using adjacent tissue sections (5 μm apart). a) HA/PEG/PCL scaffolds. b) HA scaffolds. Black arrow–osseointegration.

To investigate the correlation between the presence of macrophages/FBGCs and osseointegration, higher-resolution immunohistochemistry images were captured to allow the analysis of their distribution and morphology. Compared to the H&E staining images in Fig. 4 which clearly showed the distribution of fibrous tissue, the presence of macrophages/FBGCs were more differentiable in these immunohistochemistry images. For the HA scaffolds, a heterogeneous interface with the coexistence of FBR and osseointegration was commonly found (Fig. 8). The composite scaffolds also showed occasional direct bone-biomaterial contacts. There were no macrophages/FBGCs at osseointegration locations whilst no direct bone-biomaterial contact was seen where macrophages/FBGCs were present. However, FBR and osseointegration were found in proximity at some locations, which suggested that if an inhibitory effect by macrophages/FBGCs on bone regeneration existed it might only work at very short distance.

**Fig. 9:**
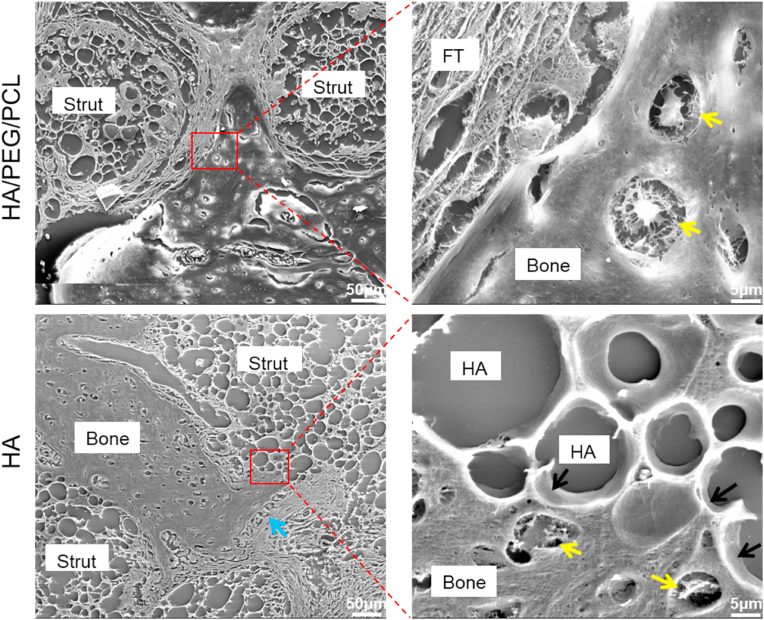
SEM images of the two different biomaterial-bone interfaces. Black arrow–osseointegration; yellow arrow–osteocyte; blue arrow–osteoblast and osteoid; FT–fibrous tissue. The particulate-shape voids in the images are where HA particles located before decalcification. Matrix was seen within the gaps between the HA particles.

To obtain a more detailed morphology of the bone-material interface, decalcified microtome-sectioned slices were images using SEM. Fibrous tissue which was commonly found in the composite scaffolds separated new bone from the strut surface (Fig. 9). In contrast, for the HA scaffolds, direct contacts between new bone and scaffold material without intervening fibrous tissue were more common. Uncalcified osteoid and osteoblasts on the surface of growing new bone were also visible (blue arrow Fig. 9), which also demonstrated the direction of bone growth towards biomaterial surface. Direct contacts between HA particles and new bone without intervening fibrous tissue were observed in the SEM images (black arrows Fig. 9). The decalcified nature of the samples as well as resolution limit of SEM inhibited further investigation of the interfacial details (e.g. epitaxy growth of bone mineral on HA particle surfaces). It would be interesting to analyse the ultrastructure of this interface with undecalcified samples in the future using transmission electron microscopy which offers even higher resolution.

## 4 Discussion

Existing literature in osseointegration lack consideration of the role of FBR in the formation of bone-biomaterial interface. Studies that have investigated the material-bone interface using high resolution electron microscopy have illustrated the ultrastructure of osseointegration. However, these studies lacked analysis on the overall status of the interface and gave an impression of a homogenous bonding across the entire material surface. Increasingly, the effect of FBG has been taken into account in considering the formation of osseointegration, e.g. for titanium dental implants [36]. However, the role of FBR in the formation of osseointegration still lacks clarity. On the other hand, FBR is a common phenomenon to all implanted biomaterials [14]. The endeavour of searching a biomaterial with no or minimum FBR and fibrosis has been approved to be very challenging [[37], [38], [39]]. Even for bone mineral-like biomaterials, such as calcium phosphates, which some literature have claimed no FBR is associated with, some studies have presented opposing evidence. For example, multinucleated cells that have been found on implanted hydroxyapatite and Bio-Oss® (deproteinized bovine bone mineral) were identified to be which of inflammatory origin instead of osteoclasts [40]. A study showed heterogeneous hydroxyapatite-human periodontal tissue interfaces (17 cases with fibrous tissue, 2 cases with HA particles encased in bone) [20]. A study which reviewed correlation between biomaterial surface characteristics and osseointegration for dental implants also showed lack of consensus on how osseointegration is related to biomaterial surface properties [41]. All these have prompted us to carry out this study in an effort to shed more light on the formation of osseointegration in the context of FBR.

We started with two hypothesises. The first was that is the bonding between new bone and material surface formed by bone growing from the materials surface or by growing towards it? Our data strongly indicates that the latter is true. The relative locations of new bone and biomaterials shown by histological and SEM images, particularly where direct contacts were about to form and osteoblasts and osteoid were visible, demonstrated the direction of new bone growth. This conclusion can be supported by other facts. For example, the early stage of inflammatory response involves acute inflammation and chronic inflammation which are dominated by polymorphonuclear cells and mononuclear cells, respectively [14,42]. Bone stem/progenitor cells are later recruited from bone marrow and periosteum to the injury site by signalling molecules including growth factors, pro-inflammatory cytokines and angiogenic factors [[43], [44], [45], [46], [47], [48]]. Haematoma collected 4 days after rat femoral trauma was found to be able to form ectopic (intramuscular) bone whilst two-day fracture haematoma did not [49], also suggesting the absence of osteogenic stem/progenitor cells within early fracture site. However, the population of the mixed cell types at a fracture site at early times post injury remains elusive. Moreover, the cell populations at bone fracture sites are dependent on the status of the primary haematoma after surgery. To further illustrate this process in vivo, we will need techniques that allow us to observe bone-material interfaces in vivo continuously or in time-lapse fashion. The monitoring of osteolineage cells in vivo in relation to immune cells at bone defect sites would be particularly interesting. The methods employed in this study only allowed us to observe the interfaces at sparse time points and on limited cell types. However, the evidence obtained in this study strongly supported the formation of osseointegration by new bone growing towards biomaterial surface.

It is worth noting that we have not investigated locations where direct contacts between existing bone and implants were formed during surgery. When an implant is screwed or press-fit into existing bone, there will be immediate bone-material contacts. Instead, we have investigated the interface between new bone and scaffolding materials where initial contact due to implantation was absent. We believe this is a better way to investigate the formation of osseointegration as it allowed us to exclude direct contacts caused by implantation procedures. However, how these implantation-induced direct contacts evolve over time in vivo is an interesting topic.

The effects of mechanical properties on the response of immune cells, particularly macrophages, and foreign body response have been reported previously. Generally speaking, stiffer materials induced more pro-inflammatory M1 macrophages whilst softer materials promoted anti-inflammatory M2 macrophages [50]. However, the effect of stiffness on macrophage polarization is more complex and depends on other properties of the materials [51]. The difference in modulus was relatively small between the HA and HA/PEG/PCL scaffolds which showed modulus in the low double digit and single digit MPa, respectively. We think that the difference in inflammatory response is mainly due to chemical composition rather than material stiffness. Our previous paper showed cell adhesion and proliferation on the HA/PEG/PCL scaffolds [21]. Cell adhesion and proliferation on HA scaffolds have also been widely reported. Therefore, the lack of osseointegration to the HA/PEG/PCL scaffolds was not likely due to the inability of these scaffolds supporting cell adhesion.

Our second hypothesis is that FBR will inhibit the formation of bonding between material surface and new bone. The locations where osseointegration can form therefore depends on the distribution of FBR components (macrophages/FBGCs and fibrous tissue). Our data showed a much stronger FBR for the composite scaffolds, which was evidenced by significantly more macrophages/FBGCs and fibrous tissue surrounding the material surfaces. There were only minimum direct contacts between the composite material and new bone. In contrast, significantly more direct contacts between material and new bone were observed for the HA scaffolds despite there was also presence of FBR components at some areas. There was a clear correlation between the presence of macrophages/FBGCs and absence of osseointegration. Our data shows that the local distribution of FBR components inhibits the formation of direct contact between material surface and new bone. This suggests that when we consider FBR and its effects, it's important to consider the intensity of this response and the local distribution of the FBR components. Negative correlation between osseointegration and FBR shown in our study suggests that minimizing FBR is a viable strategy to improve osseointegration. Immunomodulatory biomaterials using their physical and chemical cues or as carriers of inflammation-modulating drugs have been reported as means to minimizing FBR [52]. For example, alginate modified with certain functional groups have demonstrated minimal FBR in a non-human primate model [53].

Inflammation can influence bone-forming stem/progenitor cells and osteoclastogenesis. Our knowledge on the interactions between immune cells in innate and adaptive immunity and bone-forming/resorbing cells has made significant progress since the start of the field of osteoimmunology [54,55]. Implantation of biomaterials can cause responses from immune cells, which leads to FBR. Therefore, the interplay between biomaterials, immune cells and bone cells needs further investigation in the future. The similarity between multinucleated foreign body giant cells and osteoclasts also requires careful differentiation when studying immune cells surrounding biomaterials [56]. The implications of this complex interplay in bone regeneration, osseointegration and osteoinduction warrant further investigation.

## 5 Conclusions

Evidence on how direct bone-biomaterial contact is formed in vivo is sparse. The role of FBR in this process also remains elusive. This study has shed light on these two aspects. Two biomaterials which elicited FBR with different intensities exhibited drastically different material-bone interfaces. The HA/PEG/PCL composite scaffolds showed a more intensive FBR with strut surfaces surrounded mainly by fibrous tissue and minimum sign of osseointegration. In contrast, sintered HA scaffolds which showed less intensive FBR formed more direct material-bone contacts. However, the material-bone interface of the HA scaffolds was heterogenous with the coexistence of both osseointegration and FBR components. The formation of osseointegration was dependent on the distribution of FBR components. Where FBR components were present there was no osseointegration. In addition, the formation of direct biomaterial-bone contact was demonstrated to be via new bone growing towards biomaterial surface. This is different to the previous hypothesis of bone-forming stem/progenitor cells attaching to implanted biomaterial surfaces followed by new bone growing outwards. Future efforts in vivo tracking osteolineage cells and immune cells and revealing their molecular interactions will help further illustrate this process.

## Credit author statement

Conceptualization – Jing Yang, Jun Tao, Fanrong Ai, Methodology – Jing Yang, Jun Tao, Fanrong Ai, Dewei Qiu, Chuanliang Cao, Aruna Prasopthum, Zhenchang Sun, Shan Zhang, Hanwen Yang^2^, Zhiyong Xu, Investigation – Dewei Qiu, Chuanliang Cao, Shan Zhang, Hanwen Yang^2^, Zhiyong Xu, Formal analysis – Dewei Qiu, Chuanliang Cao, Shan Zhang, Hanwen Yang^2^, Zhiyong Xu, Writing – original draft – Jing Yang, Dewei Qiu, Chuanliang Cao, Writing – review & editing – Dewei Qiu, Chuanliang Cao, Aruna Prasopthum, Zhenchang Sun, Shan Zhang, Hanwen Yang^2^, Zhiyong Xu, Jun Tao, Fanrong Ai, Jing Yang, Supervision – Jun Tao, Fanrong Ai, Jing Yang.

## Declaration of competing interest

The authors declare that they have no known competing financial interests or personal relationships that could have appeared to influence the work reported in this paper.

## Data Availability

Data will be made available on request.
