# Supplementary material for: Elucidating osseointegration in vivo in 3D printed scaffolds eliciting different foreign body responses
Source: Mater Today Bio. 2023 Aug 19;22:100771. doi: 10.1016/j.mtbio.2023.100771 (PMC10477687; doi:10.1016/j.mtbio.2023.100771)
Supplement: Multimedia component 1 [file mmc1.docx]

Supplementary figures


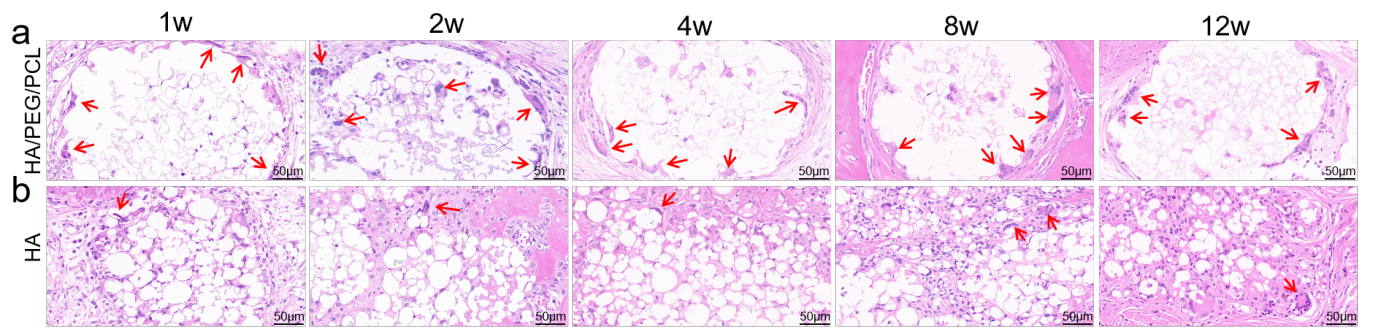


Supplementary figure 1. More FBGC associated with the composite scaffolds compared to the HA scaffolds. red arrow–FBGCs


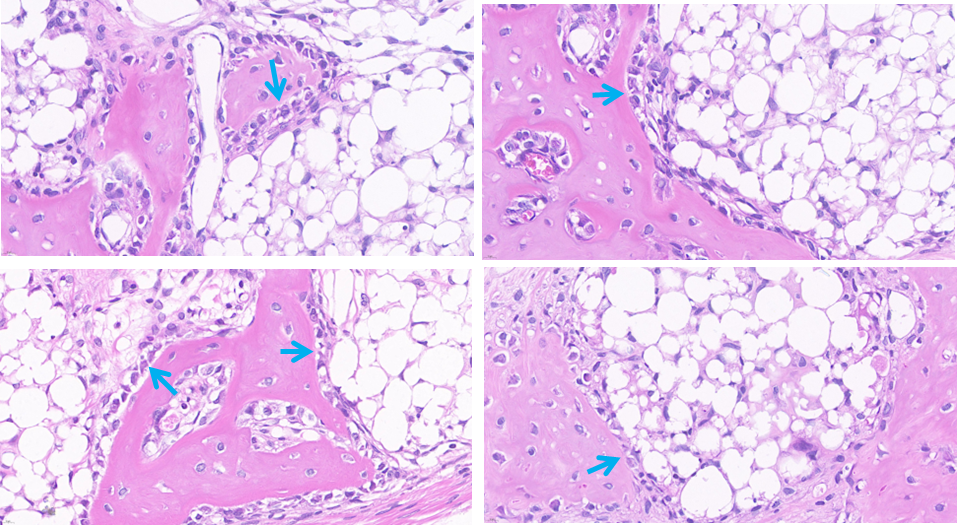


Supplementary figure 2. H&E images showing the growth of new bone towards HA particle surface. Blue arrow-osteoblasts


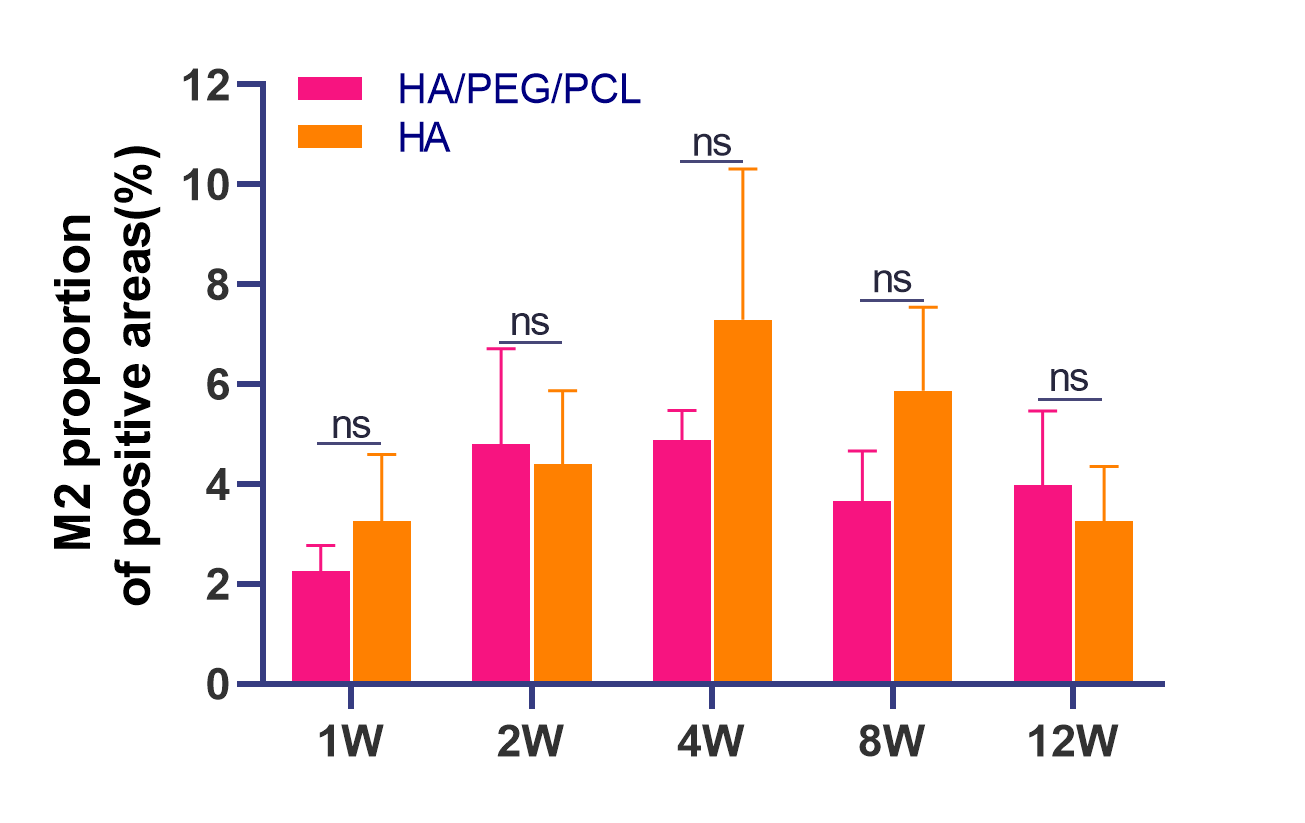


Supplementary figure 3. M2 level versus time. M2 was quantified by measuring the area that is positive for M2 marker CD163 in immunohistochemistry images.
